# Scarce evidence of the causal role of germline mutations in *UNC5C* in hereditary colorectal cancer and polyposis

**DOI:** 10.1038/srep20697

**Published:** 2016-02-08

**Authors:** Pilar Mur, Sánchez-Cuartielles Elena, Susanna Aussó, Gemma Aiza, Valdés-Mas Rafael, Marta Pineda, Matilde Navarro, Joan Brunet, Miguel Urioste, Conxi Lázaro, Victor Moreno, Gabriel Capellá, Xose S. Puente, Laura Valle

**Affiliations:** 1 Hereditary Cancer Program, Catalan Institute of Oncology, IDIBELL, 08908 Hospitalet de Llobregat, Spain; 2 Unit of Biomarkers and Susceptibility, Cancer Prevention and Control Program, Catalan Institute of Oncology, IDIBELL and CIBERESP, 08908 Hospitalet de Llobregat, Spain; 3 Department of Biochemistry and Molecular Biology, Instituto Universitario de Oncología del Principado de Asturias, Universidad de Oviedo, 33006 Oviedo, Spain; 4 Hereditary Cancer Program, Catalan Institute of Oncology, IDIBGi, 17007 Girona, Spain; 5 Department of Medical Sciences, School of Medicine, University of Girona, 17071 Girona, Spain; 6 Familial Cancer Clinical Unit, Human Cancer Genetics Program, Spanish National Cancer Research Centre (CNIO) and Center for Biomedical Network Research on Rare Diseases (CIBERER), 28029 Madrid, Spain; 7 Department of Clinical Sciences, School of Medicine, University of Barcelona, 08907 Hospitalet de Llobregat, Spain

## Abstract

Germline mutations in *UNC5C* have been suggested to increase colorectal cancer (CRC) risk, thus causing hereditary CRC. However, the evidence gathered thus far is insufficient to include the study of the *UNC5C* gene in the routine genetic testing of familial CRC. Here we aim at providing a more conclusive answer about the contribution of germline *UNC5C* mutations to genetically unexplained hereditary CRC and/or polyposis cases. To achieve this goal we sequenced the coding region and exon-intron boundaries of *UNC5C* in 544 familial CRC or polyposis patients (529 families), using a technique that combines pooled DNA amplification and massively parallel sequencing. A total of eight novel or rare variants, all missense, were identified in eight families. Co-segregation data in the families and association results in case-control series are not consistent with a causal effect for 7 of the 8 identified variants, including c.1882_1883delinsAA (p.A628K), previously described as a disease-causing mutation. One variant, c.2210G > A (p.S737N), remained unclassified. In conclusion, our results suggest that the contribution of germline mutations in *UNC5C* to hereditary colorectal cancer and to polyposis cases is negligible.

Estimates indicate that inherited factors account for over 20% of all colorectal cancer (CRC) cases, however, a relevant proportion of the genetic predisposition to CRC is not explained by germline mutations in known high-penetrance genes^1^. In the last years, advances in high-throughput genotyping and massively parallel sequencing techniques have facilitated the identification of multiple low- and high-penetrance genes responsible for the predisposition to CRC^2^,^3^,^4^,^5^,^6^,^7^,^8^,^9^,^10^,^11^,^12^. However, the identified low-penetrance alleles and high-penetrant genes only explain a small proportion of familial cases. The identification of novel CRC predisposing genes, and the validation and characterization of newly-described candidate genes are key to improve the processes of genetic testing and genetic counseling in familial cancer, by personalizing cancer surveillance and, if possible, the diagnostic and therapeutic approaches in mutation carriers.

The *UNC5C* gene encodes a protein that belongs to the UNC-5 family of netrin receptors. Netrin-1 is a diffusible laminin-related protein that plays a major role in the control of neuronal migration during nervous system development^13^. However, it has been shown to be implicated in multiple functions beyond neural development, such as the regulation of endothelial and epithelial cell survival by inhibiting the pro-apoptotic activity of the dependence receptors DCC and UNC5H, including *UNC5C*^14^,^15^,^16^.

Based on previous evidence demonstrating the role of *UNC5C* and other Netrin-1 receptors, such as DCC, as tumor suppressors and their association with intestinal tumor initiation and progression^17^,^18^,^19^,^20^,^21^,^22^,^23^,^24^, Coissieux *et al.* hypothesized that germline mutations in the *UNC5C* gene might predispose to CRC^25^. By performing a mutational screening of the whole coding region of the gene in 235 unrelated familial CRC cases, the authors identified a total of 5 novel or rare (MAF < 1%) non-synonymous -all of them missense- variants. Considering the location of the 4 predicted-to-be-functionally-relevant variants, -p.D353N, p.R603C, p.Q603E and p.A628K-, they extended the mutation screening of exon 7 (extracellular thrombospondin domain) and exons 9–11 (ZU-5 domain, involved in the pro-apoptotic activity of the protein) to 582 additional familial CRC cases, identifying 10 additional carriers of the candidate variants. Moreover, since exon 11 condensed most of the identified changes, they analyzed it in 984 additional unrelated CRC-affected individuals (aproximately 10% of them classified as familial CRC cases) but found no alterations. Of the 4 candidate variants, only p.A628K showed statistically significant differences between CRC cases and controls but only in one of the three studied populations (3/328 cases vs. 2/1911 controls). On the basis of co-segregation data in 3 families carrying p.A628K, presence of promoter methylation in the carriers’ tumors, and *in vitro* evidence of a functional effect of the variant inhibiting apoptosis, the authors suggested a causal role of *UNC5C* p.A628K in hereditary CRC. However, the fact that the association with cancer was only observed in one cohort and that the pro-apoptotic activity was also affected by the other 3 missense variants that were not associated with cancer, warranted the validation of the obtained results in independent series.

More recently, Küry *et al*.^26^ sequenced exon 11 of *UNC5C,* which contains p.A628K, in 120 familial CRC cases of unknown genetic etiology, either with mismatch repair (MMR)-deficient or proficient tumors, 35 polyposis patients, 23 patients affected with Lynch syndrome-spectrum tumors, 132 carriers of germline mutations or variants of uncertain significance (VUS) in the MMR genes, and 300 unaffected controls. Also, p.A628K and p.Q630E were genotyped in 1023 sporadic patients and 821 additional unaffected controls. The p.A628K variant was identified in a gastric cancer patient, in a Lynch syndrome patient, and in a patient carrier of a VUS in *MLH1*, for whom the *UNC5C* variant did not segregate with cancer in the family. No association of p.A628K with cancer was observed when comparing cases and controls. Two additional variants in exon 11 were identified in two patients with familial CRC of unknown etiology: p.R603C, previously reported by Coissieux *et al.* as not associated with CRC, and p.T617I, a novel variant predicted to be benign.

Here we aim at providing a more definitive answer about the role of germline mutations in *UNC5C* in the inherited predisposition to CRC by sequencing the whole coding region of the gene in a large series of familial CRC cases without mutations in known high-penetrance genes.

## Results and Discussion

**Figure 1:**
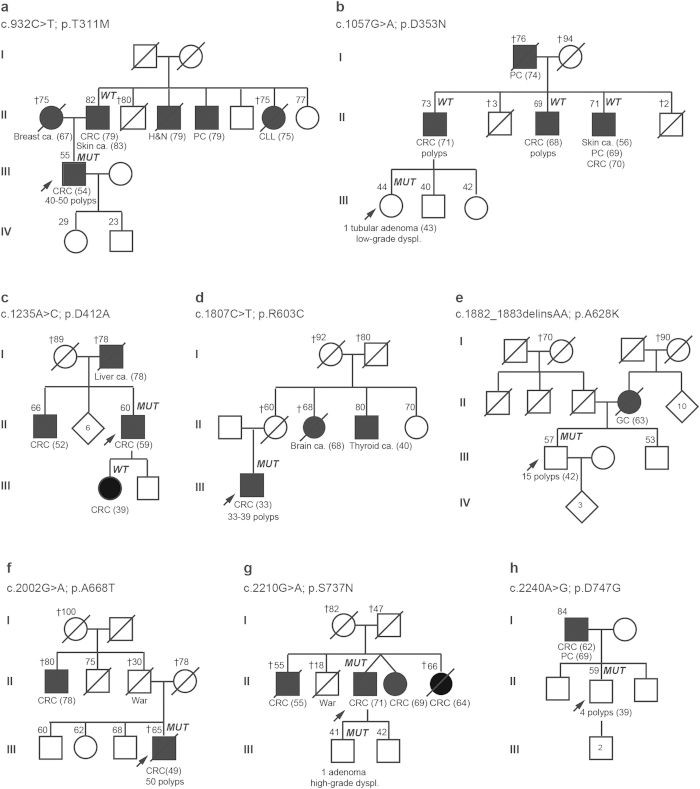
Pedigrees of the eight families with *UNC5C* variants. Filled symbol, cancer. Ages at information gathering or at death (†), when available, are indicated on the top-left corner, and ages at cancer diagnosis, between brackets after tumor type. Abbreviations: ca, cancer; dyspl, dysplasia; CLL, chronic lymphocytic leukemia; CRC, colorectal cancer; GC, gallbladder cancer; H&N, head and neck cancer; PC, prostate cancer; *MUT*, mutation carrier; *WT,* non-carrier of the mutation identified in the family.

All exons and flanking sequences of *UNC5C* (16 exons) were sequenced using a strategy that combines pooled DNA amplification and targeted-gene massively parallel sequencing in 544 familial CRC or polyposis patients (529 families) without pathogenic mutations in known high-penetrance genes. These included 456 uncharacterized MMR-proficient CRC families, 60 of them Amsterdam-positive, and 88 unrelated polyposis cases. The mutation screening revealed a total of eight novel or rare (population MAF < 1%) non-synonymous variants in *UNC5C* in eight independent families (Table 1, Fig. 1).

Co-segregation analyses definitively discarded two of the detected variants, c.1057G > A (p.D353N) and c.1235A > C (p.D412A), as the cause of CRC aggregation in the corresponding families (Fig. 1, families B and C). A third variant, c.932C > T (p.T311M), did not segregate with the paternal branch, where most of the tumors aggregated in the family (Fig. 1, family A). This, together with the lack of association with CRC in a case-control study (MCC-Spain) (Table 1), argues against a causal effect for p.T311M. Due to sample unavailability and/or scarce family cancer history of the probands, no conclusive results from the co-segregation studies were obtained in the other five carrier families. These included three attenuated polyposis cases (polyp number range: 15–50), one Bethesda-positive family, and another family with a male affected with CRC and prostate cancer at ages 62 and 69 respectively, and his son with 4 polyps, at least two of them adenomas, at age 39 (Fig. 1, families D-H).

Two of the five remaining *UNC5C* variants, c.1807C > T (p.R603C) and c.1882_1883delinsAA (p.A628K), both located in the region coding the ZU5 domain of the protein (exon 11), had been previously reported in familial CRC cases. The p.R603C variant had been identified in a patient with CRC diagnosed at age 38, polyps and familial cancer history of CRC and polyps^26^, and in two unrelated individuals with CRC (ages 56 and 58) and familial cancer history of CRC and other tumors^25^. In our study, the p.R603C mutation carrier was diagnosed with CRC and 33–39 polyps at age 33, and had a maternal aunt with a brain tumor at age 68 and a maternal uncle with thyroid cancer at age 40 (Fig. 1, family D). In none of the four reported families with p.R603C co-segregation analyses were performed. In our study, it was identified in 1 of 529 families studied (MAF: 0.09%; 1/1058), which lays below the population allele frequency described in Caucasians (MAF_Caucasians_ESP6500_: 0.21%; 18/8582). Moreover, the fact that no association with CRC was identified when comparing cases and controls^25^, suggests that this variant is not implicated in the genetic predisposition to CRC.

The other variant located in the ZU5 domain of the protein (exon 11), c.1882_1883delinsAA (p.A628K), was predicted to have a neutral effect on the protein function by computational algorithms (Table 1). It was identified in a cancer-free individual diagnosed with 15 polyps at the age of 42, whose mother had a gallbladder tumor at 63 (Fig. 1, family E). Coissieux *et al*.^25^ had identified the same variant in 5 CRC families: Co-segregation analyses performed in 3 of them was consistent with a role of p.A628K in the familial aggregation of CRC^25^. Küry *et al*.^26^ identified the same variant in three additional cancer families: One of these families carried a germline pathogenic (stop-gain) mutation in *MSH2*. In another family, where cancer segregated with a VUS in *MLH1* (p.L585A), *UNC5C* p.A628K was identified in 3 cancer-free relatives (ages 57–68) and not in a relative diagnosed with metachronous CRC at age 41 and 55. In all, the evidence gathered discards the role of p.A628K in the predisposition to CRC in the family. In the third family, characterized by the presence of gastric tumors, *UNC5C* p.A628K was detected in the two individuals with gastric cancer studied^26^. If p.A628K increased risk for CRC, as previously suggested by Coissieux *et al*.^25^, the variant would occur more frequently in cases than in controls. This finding was observed once in a French cohort (3/328 cases vs. 2/1911 controls (p = 0.031)), but never confirmed in three additional series^25^,^26^, including an independent French study^26^. Compiling all 4 series studied, no statistically significant difference in the frequency of p.A628K in CRC cases (9/2824) and controls (7/5265) was identified (p-value = 0.1115) (Table 1).

Of the three remaining families, no conclusive results were obtained from the family information for c.2002G > A (p.A668T) and c.2240A > G (p.D747G) (Fig. 1, families F and H). However, the results of a case-control study suggest that they do not associate with an increased risk to CRC (Table 1), although larger case-control series should be studied to reach a desirable statistical power.

Finally, the c.2210G > A (p.S737N) variant was identified in an individual diagnosed with CRC at age 71 and in his son, who had a villous adenoma with high-grade dysplasia at age 41. The proband had three brothers and sisters affected with CRC (ages 55–69), but sample unavailability prevented us from studying their carrier status (Fig. 1, family G). *In silico* prediction of the effect of the amino acid change on the protein function was also inconclusive, although the affected amino acid is relatively well conserved in evolution (Table 1). The variant had not been reported in public databases and was not present in over 800 alleles of Spanish origin (in-house data). The evidence gathered so far does not allow us to discard or confirm the causal role of the variant on the predisposition to CRC. Functional analyses are often key to determine the role of unclassified variants, albeit, to our knowledge, for this case there are not currently available standardized assays.

The identification of second inactivating somatic hits provides additional evidence supporting a pathogenic role for a variant of unknown significance in a given family. It had been previously suggested that the wild-type allele of *UNC5C* was silenced through promoter methylation in the tumors developed by carriers of *UNC5C* variants. However, this methylation of the promoter region is a common feature in the majority of colon tumors^23^,^27^,^28^,^29^, independently of the inactivation of the other allele, either germline or somatic. Access to CpG methylation data from paired tumor and normal adjacent colon mucosa from 92 sporadic cancer patients revealed that all colon tumors showed increased methylation levels in the CpGs of the promoter region compared to their paired normal colon mucosae (Supplementary Figure 1). Specifically, 83% (76/92) of tumors showed a difference of over 20% in the methylation levels of the promoter CpGs compared to their normal counterparts. We identified monoallelic CpG methylation in the *UNC5C* promoter in all the tumors developed by carriers of *UNC5C* variants that were analyzed (6 tumors from 5 families) (Supplementary Table 1), as well as in all sporadic CRC cases that were included in the analyses (n = 7). However, we could not determine which allele was methylated due to the absence of informative markers in the studied region. On the other hand, no LOH of the *UNC5C* locus was detected in 4 of 5 the tumors studied that were developed by *UNC5C* variant carriers (Supplementary Table 1). In all, *UNC5C* somatic analysis was of no help to further classify the identified variants.

Co-segregation studies in the families are key to support or discard the causal role of rare variants. This has been the case for three families carrying *UNC5C* variants identified in this study, where co-segregation data indicated or strongly suggested absence of causal effect for c.1057G > A (p.D353N), c.1235A > C (p.D412A) and c.932C > T (p.T311M). Similarly, absence of segregation of c.1882_1883delinsAA (p.A628K) with cancer was observed in one family studied by Küry *et al*^26^. Not only in the research scenario for the identification and/or validation of novel predisposing genes, but also in the routine diagnostics of hereditary cancer for the characterization of variants of uncertain significance in known genes, co-segregation studies are crucial for the precise classification of variants, bringing up the need to routinely collect samples from affected and non-affected family members in a comprehensive manner. Unfortunately, it is becoming increasingly difficult to obtain meaningful co-segregation information due to the size of family pedigrees and to the access to relevant biological samples.

Data from CRC case-control studies for the identified *UNC5C* variants were available for 6 of the 8 identified variants (previously reported data and/or our own) (Table 1), showing lack of association with CRC. However, the power of this study to detect an association was low due to the rarity of the variants. To illustrate this caveat and considering 0.05% a representative MAF for the studied variants, 47,000 cases and 47,000 controls would be required to detect an OR > 2 (moderate to high penetrance) with a power of 80%. These numbers increase to 118,000 or 236,000 cases and controls if the MAF is 0.02% or 0.01%, respectively. In the case of c.1882_1883insdelAA (p.A628K), with a MAF observed in the genotyped controls of 0.13% (7/5265), the sample size required for the above mentioned conditions would be 18,000 cases and 18,0000 controls. Moreover, since this variant has not been reported in public databases, it is not included in the genotyping arrays used for genome-wide association studies, and being an indel, it is not reported in publicly available exome sequencing results, making it even more difficult to gather the required sample size.

In conclusion, we have identified a total of 8 rare or novel *UNC5C* variants in 529 unexplained CRC families and polyposis cases. Evidence gathered in the families and in CRC case-control studies, although underpowered, suggests that at least 7 of these 8 variants do not cause increased CRC risk, including *UNC5C* c.1882_1883delinsAA (p.A628K), previously described as pathogenic. Therefore, our findings, together with previous evidence, suggest that the contribution of germline mutations in *UNC5C* to CRC predisposition is likely non-existent or extremely rare.

## Methods

### Patients

A total of 544 CRC cases including 456 non-polyposis familial and/or early onset CRC patients and 88 individuals with polyposis were examined^11^. Patients were assessed in Spain at the Genetic Cancer Counseling Units of the Catalan Institute of Oncology and the Spanish National Cancer Research Center (CNIO), between 1999 and 2012. Informed consent was obtained from all subjects and the study received the approval of the Ethics Committee of the Institut d’Investigació Biomedica de Bellvitge (IDIBELL) (PR073/12). The methods were carried out in accordance with the approved guidelines.

### DNA and RNA extractions

Genomic DNA from peripheral blood was extracted using the FlexiGene DNA kit (Qiagen) and from formalin-fixed paraffin-embedded samples, using the QIAamp DNA FFPE tissue Kit (Qiagen, Hilden, Germany), both following manufacturer’s instructions. RNA from cultured lymphocytes was extracted using a standard Trizol-based protocol.

### Mutation identification in pooled samples

Patients were screened for *UNC5C* mutations using a combination of PCR amplification in pooled DNAs and targeted massively parallel sequencing as previously described^30^. Amplification of the DNA pools was performed with the Phusion High-Fidelity DNA Polymerase (New England Biolabs, Ipswich, MA, USA) and custom-designed primers (Supplementary Table 2). Equimolar amounts of each amplicon were pooled, ligated and fragmented using a Covaris S2 (Covaris, Inc. MS, USA), and DNA libraries were prepared following the paired-end sample preparation protocol from Illumina (Illumina, Inc. CA, USA). Next generation sequencing was carried out on a HiSeq-2000 at the Centro Nacional de Análisis Genómico (CNAG, Barcelona, Spain). Variant identification was performed as previously described^30^ and common variants present in dbSNP141 or 1000Genomes with a population minor allele frequency higher that 1%, were considered polymorphisms.

### Direct automated sequencing

Sanger sequencing of the affected exon was performed to identify the mutated individuals among the samples included in the corresponding DNA pool, and for the co-segregation studies in the families. Sequencing was performed on an ABI Sequencer 3730 using a standard protocol and data was analyzed with Mutation Surveyor v.3.10. Primer sequences are shown in Supplementary Table 2.

### *In silico* predictions

The impact of missense variants at the protein level was analysed using the *in silico* algorithms SIFT, PolyPhen-2, Mutation Taster and CONDEL^31^,^32^,^33^,^34^. The potential effects on splicing were evaluated by using Human Splice Finder v.3.0^35^. Prediction data from SIFT, PolyPhen-2 and Human Splice Finder v.3.0 were provided by Alamut Visual v2.7.1 software (Interactive Biosoftware, Rouen, France). PhyloP and PhastCons conservation scores were obtained from the Mutation Taster website (www.mutationtaster.org).

### Culture of lymphocytes and splicing analysis

The alteration of splicing by the change *UNC5C* c.932C > T was evaluated using peripheral blood lymphocytes from the corresponding carriers cultured in presence and absence of puromycin^36^. Synthesis of cDNA from the extracted RNA was performed using Transcriptor First Strand cDNA Synthesis Kit (Roche Diagnostics GmbH, Mannheim, Germany). PCR amplification was performed for the region comprised between exons 4–8 of the *UNC5C* gene. PCR products were run in a 1.5% agarose gel, visualized in a UV transilluminator and subsequently sequenced. The primers sequences used are detailed in Supplementary Table 2.

### Loss of heterozygosity (LOH)

A set of three microsatellite markers (D4S1559, D4S2380 and D4S470) covering the chromosome region 4q21-23 was used to determine LOH in *UNC5C*. PCRs were performed on DNA extracted from paraffin-embedded tumor tissue and compared with DNA extracted from normal colon tissue or peripheral blood. Primer sequences are detailed in Supplementary Table 2.

### Promoter methylation analysis

One μg of genomic DNA was subjected to bisulfite treatment using the EZ DNA Methylation-Gold Kit (Zymo Research, Orange, CA, USA). A total of 1 μl of bisulfite-converted DNA was used in two PCR reactions for the amplification and subsequent sequencing of *UNC5C* promoter region (−396 to −60; 23 CpG sites) (Supplementary Figure 2). In order to identify tumor-specific promoter methylation, we compared bisulfite sequencing results between tumor and normal tissue (blood or normal colon mucosa) of the same patient. Primer sequences are shown in Supplementary Table 2.

### Validation studies on sporadic CRC

The association of the *UNC5C* variants with sporadic CRC was assessed in a population-based multicase-control study (MCC-Spain, www.mccspain.org)^37^. This included 1336 CRC patients and 2744 cancer-free controls from Spain genotyped with the Illumina Infinium HumanExome BeadChip array that essentially includes rare variants in coding regions observed in the 1000 Genomes project. Some of the variants identified in our families were not included in the array. The genotyping arrays were called with the CHARGE Consortium cluster file and quality control followed their recommended criteria^38^. Subjects with ethnic origin far from the CEU HapMap cluster in the principal components analysis were excluded.

The promoter methylation analyses of colon mucosa were done in samples from 100 sporadic colon cancer patients (tumor and paired adjacent mucosa) and 50 healthy colon mucosa samples donated at colonoscopy (COLONOMICS project^39^, www.colonomics.org). These samples were analyzed with the Illumina Infinium HumanMethylation450 BeadChip array.

### Statistical analyses

Fisher’s exact test was used to evaluate genotype frequencies between cases and controls, both for the analysis of results obtained in the MCC-Spain study and for the combined analysis of reported cases and controls genotyped for c.1882_1883delinsAA (p.A628K).

## Additional Information

**How to cite this article**: Mur, P. *et al.* Scarce evidence of the causal role of germline mutations in *UNC5C* in hereditary colorectal cancer and polyposis. *Sci. Rep.* **6**, 20697; doi: 10.1038/srep20697 (2016).

## Supplementary Material

Supplementary Information

## Figures and Tables

**Table 1:** Novel and rare (population MAF < 1%) germline *UNC5C* variants identified in 529 CRC and/or polyposis families.

aVariant [protein domain]	Population MAF% (1000G/ESP/ExAC)	Protein function prediction (score)	bEvolutionary conservation (PhyloP /PhastCons)	cSplice-site prediction	Proband’s phenotype	Familial cancer history (Fig. 1)	Criteria	Co-segregation analysis	CRC cases vs. controls (MCC-Spain)	CRC cases vs. controls (Coissieux *et al*.)^25^
c.932C > T (p.T311M) [Thrombospondin]	rs200437262 (-/0.04/0.012)	dPPH2: PrD (0.983)/PsD (0.765) SIFT: D (0.02) Mut. taster: D Condel: D (0.531)	5.987/0.976	Creates ESS and disrupts ESE RNA study: no change	CRC (54), 40-50 polyps	Fam A: CRC (79) and SC (83), BC (67), H&N ca. (79), PC (79), CLL (75)	Attenuated polyposis	Non-carriers: Father (CRC 79, and SC 83), daughter (unaffected, 29)	2/1334 vs. 1/2740 (p = 0.251)	n.a.
c.1057G > A (p.D353N) [Thrombospondin]	rs145155041 (0.14/0.15/0.15)	PPH2: PsD (0.613)/N (0.166) SIFT: N (0.15) Mut. Taster: D Condel: N (0.492)	3.145/0.954	Disrupts ESE	Tubular adenoma with low-grade dysplasia (43)	Fam B: CRC (71), CRC (68), CRC (70), SC (56), PC (69)	Bethesda	Non-carriers: Father (CRC, 71)	12/1334 vs. 24/2742 (p = 1)	5/755 vs. 16/2740 (p = 0.791)
c.1235A > C (p.D412A) [Cytoplasmic domain]	-(-/-/0.002)	PPH2: PrD (0.999/0.994) SIFT: N (0.09) Mut. Taster: D Condel: D (0.611)	5.127/1	Disrupts ESE	CRC (59)	Fam C: CRC (52), CRC (39)	Amsterdam	Non-carrier: Daughter (CRC, 39)	n.a.	n.a.
c.1807C > T (p.R603C) [ZU5]	rs139568380 (0.04/0.15/0.093)	PPH2: PrD (1/0.975) SIFT: D (0) Mut. Taster: D Condel: N (0.467)	1.628/1	No change	CRC (33), 33-39 polyps	Fam D: Brain ca. (68), TC (40)	Attenuated polyposis	n.a.	n.a.	2/1801 vs. 9/4044 (p = 0.521)
c.1882_1883delinsAA (p.A628K) [ZU5]	-	PPH2: N (0.042/0.042) SIFT: N (0.87) Mut. Taster: D Condel: n.a.	1.733/0.24 −0.359/0.217	No change	15 polyps (42)	Fam E: Gallbladder ca. (63)	Attenuated polyposis (<20 polyps)	n.a.	n.a.	5/1801 vs. 5/4144 (p = 0.182); e4/1023 vs. 2/1121 (p = 0.434); TOTAL: 9/2824vs. 7/5265(p = 0.111)
c.2002G > A (p.A668T) [Cytoplasmic domain]	rs187196396 (0.02/ -/0.005)	PPH2: PsD (0.813)/N (0.083) SIFT: N (0.64) Mut. Taster: D Condel: D (0.536)	1.304/0.999	No change	CRC (49), 50 polyps	Fam F: CRC (78)	Attenuated polyposis	n.a.	2/1336 vs.2/2743 (p = 0.601)	n.a.
c.2210G > A (p.S737N) [Cytoplasmic domain]	-	PPH2: PrD (0.981)/PsD (0.843) SIFT: N (0.64) Mut. Taster: D Condel: N (0.461)	3.545/1	No change	CRC (71)	Fam G: CRC (55), CRC (69), CRC (64)	Bethesda	Carrier: Son (villous adenoma with high-grade dysplasia, 41)	n.a.	n.a.
c.2240A > G (p.D747G) [Cytoplasmic domain]	rs146792764 (0.04/0.007/0.015)	PPH2: PrD (0.983/0.982) SIFT: D (0.04) Mut. Taster: D Condel: D (0.582)	3.492/0.995	Creates donor site and disrupts ESE	4 polyps (39)	Fam H: CRC (62), PC (69)	No criteria	n.a.	3/1336 vs.5/2744 (p = 0.722)	n.a.

Characteristics of the variants and carrier families, and results of association studies in CRC patients and controls.

^a^RefSeq GRCh37: *UNC5C*, NM_003728; NP_003719.

^b^PhyloP score (values between −14 and +6): Sites predicted to be conserved are assigned positive scores. PhastCons score (values between 0–1): It reflects the probability that each nucleotide belongs to a conserved element, based on the multiple alignment of genome sequences of 46 different species (the closer the value is to 1, the more probable the nucleotide is conserved).

^c^Prediction obtained from the Human Splicing Finder (http://http://www.umd.be/).

^d^Polyphen-2: HumDiv/HumVar scores.

^e^Küry *et al.* (2014)^26^.
