# Erratum: Scarce evidence of the causal role of germline mutations in *UNC5C* in hereditary colorectal cancer and polyposis

**DOI:** 10.1038/srep23880

**Published:** 2016-04-20

**Authors:** Pilar Mur, Elena Sánchez-Cuartielles, Susanna Aussó, Gemma Aiza, Rafael Valdés-Mas, Marta Pineda, Matilde Navarro, Joan Brunet, Miguel Urioste, Conxi Lázaro, Victor Moreno, Gabriel Capellá, Xose S. Puente, Laura Valle

Scientific Reports 6: Article number: 2069710.1038/srep20697; published online: 02082016; updated: 04202016.

The original version of this Article contained errors in the spelling of the authors Elena Sánchez-Cuartielles and Rafael Valdés-Mas which were incorrectly given as Sánchez-Cuartielles Elena and Valdés-Mas Rafael respectively. These errors have now been corrected in the PDF and HTML versions of the Article.

