# Supplementary material for: Scarce evidence of the causal role of germline mutations in UNC5C in hereditary colorectal cancer and polyposis
Source: Sci Rep. 2016 Feb 8;6:20697. doi: 10.1038/srep20697 (PMC4745060; doi:10.1038/srep20697)
Supplement: Supplementary Information [file srep20697-s1.pdf]

SUPPLEMENTARY MATERIAL

Scarce evidence of the causal role of germline mutations in *UNC5C* in hereditary colorectal cancer and polyposis

Pilar Mur[1,+], Elena Sánchez-Cuartielles[1,+], Susanna Aussó[2], Gemma Aiza[1], Rafael Valdés-Mas[3], Marta Pineda[1], Matilde Navarro[1], Joan Brunet[4,5], [6]Miguel Urioste[6], Conxi Lázaro[1], Victor Moreno[2,7], Gabriel Capellá[1], Xose S. Puente[3], Laura Valle[1,*].

**lvalle@iconcologia.net*

+These authors contributed equally to this work.

Supplementary Table 1. LOH and methylation results for *UNC5C.*

**Germline mutation**[a]	**Family ID** (Fig. 1)	**Index patient**	**LOH (informative markers)**	**Promoter methylation**
c.932C>T (p.T311M)	A	III.1	n.a.	n.a.
c.1057G>A (p.D353N)	B	III.1	No LOH (D4S2380, D4S470, D4S1559)	Yes
c.1235A>C (p.D353N)	C	II.8	No LOH (D4S2380, D4S470, D4S1559)	Yes
c.1807C>T (p.R603C)	D	III.1	n.a.	Yes
c.1882_1883delinsAA (p.A628K)	E	III.1	No LOH (D4S2380, D4S470, D4S1559)	n.a.
c.2002G>A (p.A668T)	F	III.4	n.a.	n.a.
c.2210G>A (p.S737N)	G	II.3 and III.1	No LOH (D4S2380, D4S470, D4S1559)	Yes
c.2240A>G (p.D747G)	H	II.2	No LOH (D4S2380)	Yes

[a]RefSeq GRCh37: UNC5C, NM_003728
Abbreviations: LOH, loss of heterozygosity; n.a., not available information; n.i., not informative

Supplementary Table 2. Primers used in the study and experimental conditions.

	Forward (5'-3')	Reverse (5'-3')	Amplicon size (bp)	Annealing Temp.
Mutation screening				
Exon 1	TCAAACTCCTCCTCGGCGTC	GGCCGCGGAGCTTGGCGGAC	349	58C
Exon 2	ATGTGTTAACCATCTTCCAC	GTGAATCTTGAAGTGCAATG	301	58ºC
Exon 3	AGTATGAATCTTGTGTTGAC	TGGGATTACAGGCGTGAGTC	334	58ºC
Exon 4	GCACAGATAAAATACTAAGCCAGAAA	CATACCCTAATTCACTGCACCA	298	56ºC
Exon 5	ATCAGGGGATCCATGCTGAG	TGAACTAGATTGAGACCCTG	264	58ºC
Exon 6	GTGTTGTGCATGCACACATG	GGAGATACTTGGAATGAGAG	257	58ºC
Exon 7	CTCCATCCCTTAAGCAGCTG	CCCTCAATGTCTGCAGTTTG	260	58ºC
Exon 8	TATAAGCAGCTGGTTCAATG	AAGTAACAGGGTGTGAAGTG	264	58ºC
Exon 9a	CTGAATGGAGAATGCCAATG	GACAGCTTGGACGTAAACTC	250	58ºC
Exon 9b	CAAAGTGTACAACACCTCAG	AATATGTTTCAAGCTGCCTC	256	58ºC
Exon 10	TCATTCCCTTTTACGTGCTG	CATTGGTAAAGTCAGTGCAC	186	58ºC
Exon 11	ACCCAAGTGCATAGTTCATG	TGCTCCTGCTTATCTGCATG	296	58ºC
Exon 12	TCCTGGGTGACTTAGCCATG	TTCCAGGTGGAGGTGAAGAG	400	58ºC
Exon 13	GAGAGCTGACAGAAGTGATC	TCTTAGAGATGTCTCCAGAC	255	58ºC
Exon 14	GGCAGCAGACCATCTGAGAG	TTCCTGAGTGTCGCACTCTC	288	58ºC
Exon 15	TGTCCTCAGAAATAATGCTG	ACAGAAAGAAGCTAGTCTTG	262	64ºC
Exon 16	TAAGTCACACTGTTGAGCAC	TCCTTCATTTCCCCTTCCAG	236	58ºC
Splicing analysis				
c.932C>T (exons 4-8)	CAGGAAGTCTTACTCCAGTGTCG	AACATCATCTGAATCAGGAGCA	588	60ºC
LOH				
D4S1559	FAM_CTTCTCCAGTTTTTTTCTGAAATTGC	AGAAATAAATGCGTTGAAACCATCC	165	55ºC
D4S2380	FAM_ACCTTATTGTGCTATGGAAACA	TCAGCCAAGATTACCTACCG	195	55ºC
D4S470	FAM_ATTCTTGGAAGTCACAAACACTACA	GGTTTAGGCAGCAGTCATTTTCAA	156	55ºC
Methylation				
UNC5C_1	GGTGTTTGTGTGTGTTTTTTATAGG	TTTAAAAATCCCTCTTTCCCAATAC	204	56ºC
UNC5C_2	GTATTGGGAAAGAGGGATTTTTAAA	AACCCCACTAAACAAAAACTAAATC	157	56ºC

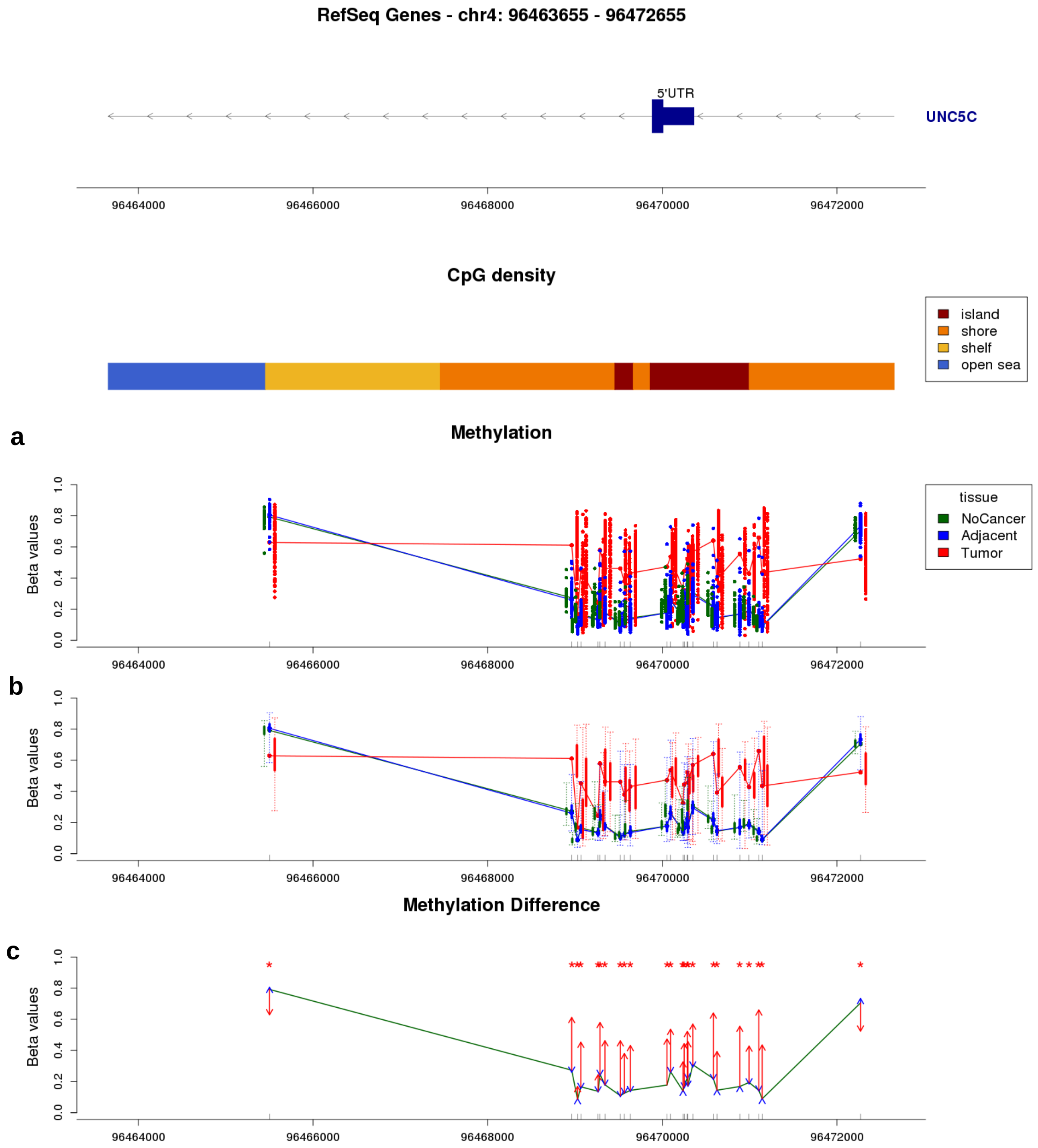

Supplementary Figure 1. CpG methylation levels in the promoter region of UNC5C in 50 normal colon tissues from cancer-free individuals (green), and in 92 colon tumors (red) and 92 paired normal mucosae (adjacent to the tumor, blue) from sporadic colon cancer patients (with no microsatellite instability). Data obtained from the Illumina Human Methylation Beadchip 450K array. a) Methylation level results represented as stripchart where each dot corresponds to one sample. b) Methylation level results represented as boxplots. C) Methylation differences between tumor and adjacent normal tissue, taking the normal mucosae as reference. Asterisks indicate statistically significant differences (t-test beta with Bonferroni correction). Complete dataset unpublished (in-house data). Almost identical results are obtained when analyzing the publicly available TCGA (The Cancer Genome Atlas) data.

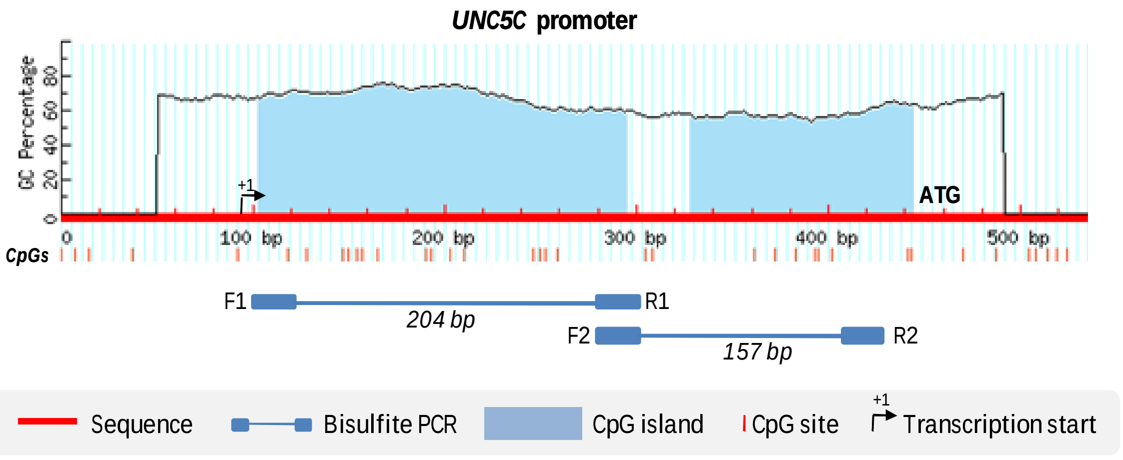

Supplementary Figure 2. Map of the CpG islands (in blue) and CpG sites (red bars) encompassing the *UNC5C* promoter (adapted from MethPrimer program), location of the primers used in the study (blue rectangles) and size of the PCR products. The translation start site of *UNC5C* is indicated by +1.
